# Supplementary material for: The Cultivation of Chelidonium majus L. Increased the Total Alkaloid Content and Cytotoxic Activity Compared with Those of Wild-Grown Plants
Source: Plants (Basel). 2021 Sep 21;10(9):1971. doi: 10.3390/plants10091971 (PMC8467419; doi:10.3390/plants10091971)
Supplement: Supplementary file 1 [file plants-10-01971-s001.zip › plants-1363476-supplementary.pdf]

Supplementary data to :

The Cultivation of *Chelidonium majus* L. Increased the Total Alkaloid Content and Cytotoxic Activity Compared with Those of Wild-Grown Plants

Supplementary Table S1. Content of alkaloids (μg/g of dry material) in aqueous ethanol extracts of wild and cultivated C. *majus* specimen

Sample	Sanguinarine	Chelerythrine	Chelidonine	Coptisine[1]	Berberine[1]	Allocryptopine[1]
**Wild 2019**						
CHM01	1.6	3.9	77.6	144.5	11.7	7.8
CHM02	0.2	2.8	19.8	172.9	12.8	5.8
CHM03	1.4	5.7	69.1	162.1	16.8	6.2
CHM04	0.8	2.8	39.8	81.6	5.6	6.1
CHM05	5.4	2.4	111.5	131.4	<LOD	<LOD
Average:	**1.9**	**3.5**	**63.6**	**138.5**	**11.7**	**6.5**
SD:	2.1	1.3	35.4	35.6	4.6	0.9
**Cultivated 2020**						
CHM01	12.1	25.7	215.0	132.3	15.4	18.2
CHM02	10.5	20.2	217.0	169.5	21.7	16.0
CHM03	9.8	11.9	105.3	114.4	9.1	9.3
CHM04	12.7	23.8	255.0	116.0	17.4	16.0
CHM05	19.0	5.7	468.4	185.2	0.2	<LOD
Average:	12.8	17.5	252.1	143.5	12.8	14.9
SD:	3.6	8.5	133.2	32.2	8.4	3.8

[1] Calculated as chelidonine

Supplementary Table S2. Content of flavonoid (µg/g of dry material) in aqueous ethanol extracts of wild and cultivated C. *majus* specimen

Sample	Kaempferol	Isorhamnetin	Quercitrin	Isorhamnetin 3-O-rutinoside	Kaempferol 3-O-rutinoside	Quercetin-3-O-rutinoside	Quercetin 3-O-galactoside	Kaempferol glucoside[1]
**Wild 2019**								
CHM01	3.90	2.88	0.89	1407	286	2730	38.81	27.11
CHM02	28.38	20.43	3.32	2834	1263	4966	657.40	336.74
CHM03	12.79	7.52	1.86	1637	726	3472	309.04	199.70
CHM04	9.98	6.27	0.39	1008	561	1825	56.22	53.98
CHM05	10.53	7.01	0.54	1177	433	2044	39.71	62.07
Average:	13.12	8.82	1.40	1613	654	3007	220.23	135.92
SD:	9.15	6.74	1.22	722	377	1270	269.91	130.77
**Cultivated 2020**								
CHM01	5.55	3.02	1.12	1461	274	3517	74.95	55.93
CHM02	6.91	5.15	2.80	2174	706	5539	330.99	61.14
CHM03	13.99	6.27	3.13	1824	802	5690	300.87	68.25
CHM04	4.39	2.96	1.50	1630	490	3241	158.50	35.00
CHM05	3.53	2.82	1.65	2196	731	3938	114.42	47.02
Average:	6.87	4.05	2.04	1857	601	4385	195.95	53.47
SD:	4.18	1.57	0.87	326	216	1151	113.95	12.90

[1] calculated as Luteolin-7-0-glucoside

Supplementary Table S3. Collection site and geographical coordinates of *Chelidonium majus* populations

Population	Municipality	Latitude	Longitude	Altitude, m
CHM01	Pededze	57.4408	27.32869	141
CHM02	Mērnieki	57.84682	24.4897	24
CHM03	Ainaži	57.86635	24.35775	46
CHM04	Viļaka	57.17996	27.6809	93
CHM05	Sigulda	57.15394	24.89847	98

Supplementary Table S4. MRM parameters applied for the analysis of alkaloids in C. *majus* extracts

Compound	MS/MS	Cone, V	Collision energy, eV
Chelerythrine	348 → 303.94, 348 → 332.94	30	20
Chelidonine	*354 → 275,* 354→ 305	30	20
Sanguinarine	332 →273.97, 332 →303.96, 332 → 316.93	30	20
Coptisine[1]	320 → 291.98, 320 → 317.97	30	20
Berberine[1]	336 → 291.99, 336 → 321	30	20
Allocryptopine[1]	370 → 188, 370→352.01	30	20

[1] Parameters set tentative

Supplementary Table S5. MRM parameters applied for the analysis of flavonoid glycoside in C. *majus* extracts

Test compound	MRM transition	Cone voltage, V	Collision energy, eV
Kaempferol	287→121, 287→165	25	30
Isorhamnetin	317→153, 317→229	30	30
Quercitrin	449→303	30	25
Isorhamnetin-3-O-rutinoside	625→317	25	20
Kaempferol 3-rutinoside	595→287	30	15
Quercetin 3-O-rutinoside	611→303	30	20
Quercetin 3-O-galactoside	465→303	30	15
Luteolin 7-O-glucoside	449→287	30	15
Reserpine (IS)	609→195	50	35

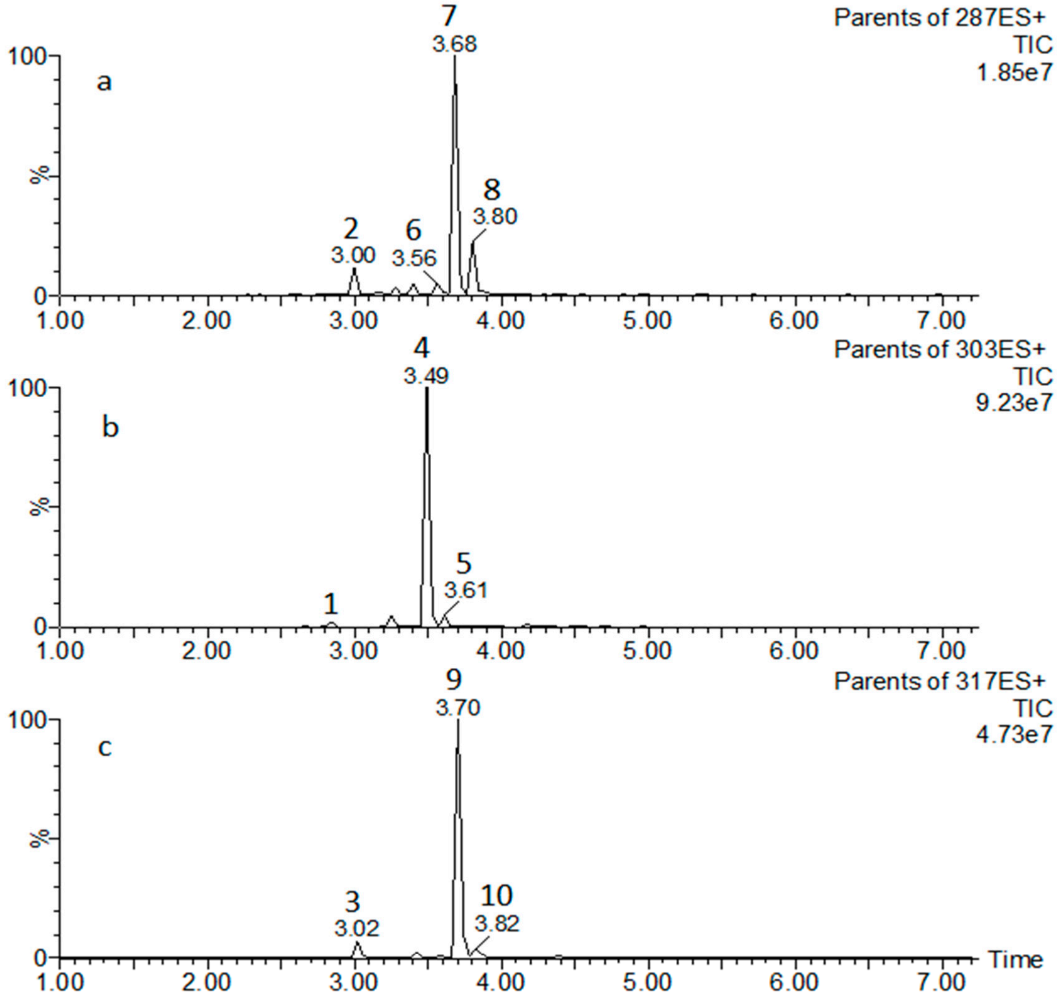

Supplementary Figure S1. Flavonoid aglycone extracted mass chromatograms (parent search): kaempferol (a), quercetin (b), isorhamnetin (c). Peak identification in Table 3.

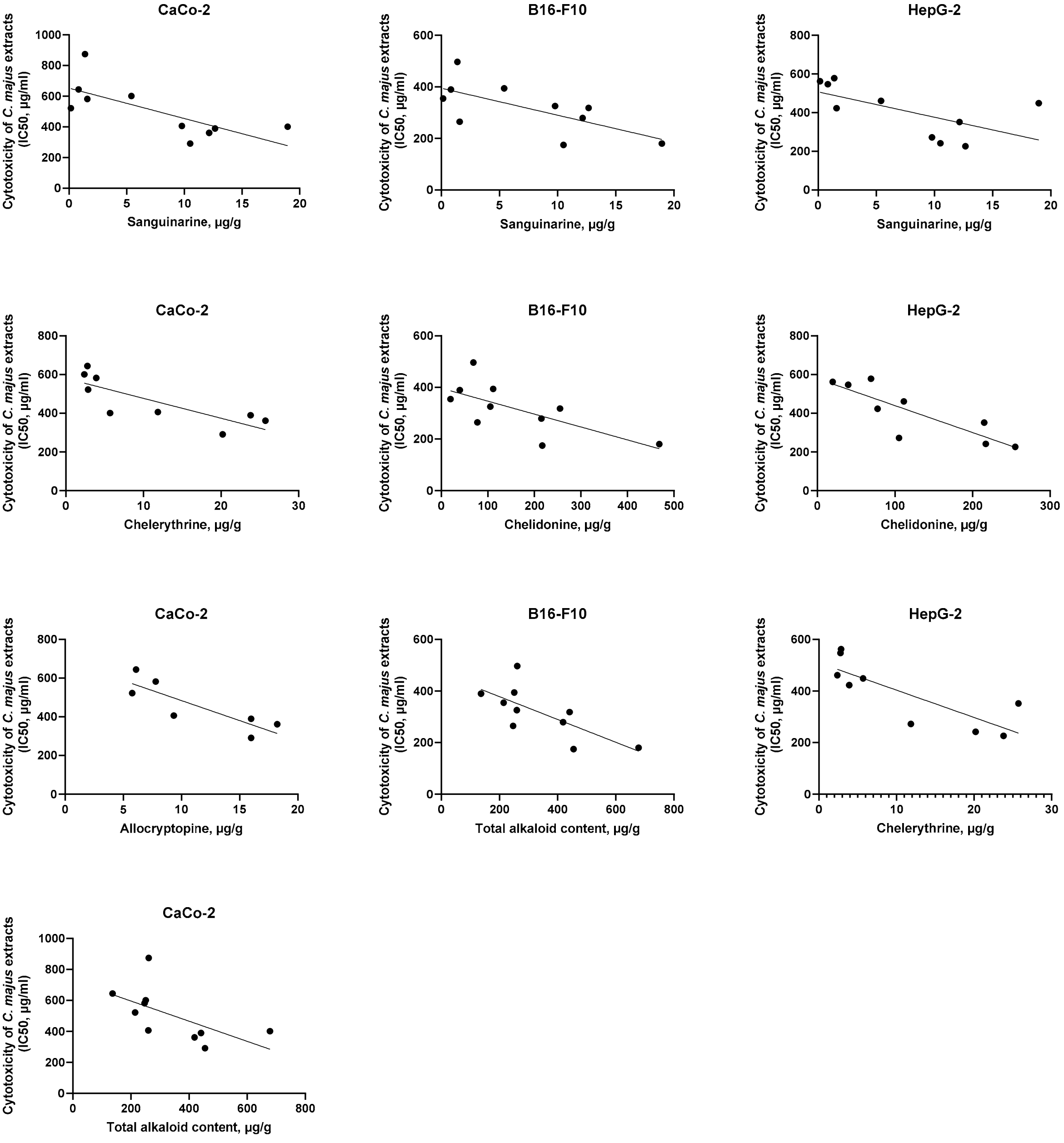

Supplementary Figure S2. Correlations between IC_{50} values of cytotoxic activities of *C. majus* extracts measured by MTT assay in three cell lines (CaCo-2, B16-F10 and HepG-2) and concentration of the alkaloids identified in the extracts by liquid chromatography – tandem mass spectrometry. The data were analyzed by the Pearson's correlation test using GraphPad Prism 8.0. Statistically significant correlations are shown in the figure. A value of $p < 0.05$ was considered to be statistically significant.
